# Silicon Nitride Background in Nanophotonic Waveguide Enhanced Raman Spectroscopy

**DOI:** 10.3390/ma10020140

**Published:** 2017-02-08

**Authors:** Ashim Dhakal, Pieter Wuytens, Ali Raza, Nicolas Le Thomas, Roel Baets

**Affiliations:** 1 Photonics Research Group, INTEC Department, Ghent University/IMEC, Gent 9000, Belgium; pieter.wuytens@ugent.be (P.W.); ali.raza@ugent.be (A.R.); nicolas.lethomas@ugent.be (N.L.T.); roel.baets@ugent.be (R.B.); 2 Center for Nano- and Biophotonics, Ghent University, Gent 9000, Belgium; 3 Phutung Research Institute, Balaju-16, Kathmandu 44611, Nepal; 4 Department of Molecular Biotechnology, Ghent University, Gent 9000, Belgium

**Keywords:** integrated optics, Raman spectroscopy, optical sensing and sensors, silicon nitride, dielectric channel waveguides, background shot noise

## Abstract

Recent studies have shown that evanescent Raman spectroscopy using a silicon nitride (SiN) nanophotonic waveguide platform has higher signal enhancement when compared to free-space systems. However, signal-to-noise ratio from the waveguide at a low analyte concentration is constrained by the shot-noise from the background light originating from the waveguide itself. Hence, understanding the origin and properties of this waveguide background luminescence (WGBL) is essential to developing mitigation strategies. Here, we identify the dominating component of the WGBL spectrum composed of a broad Raman scattering due to momentum selection-rule breaking in amorphous materials, and several peaks specific to molecules embedded in the core. We determine the maximum of the Raman scattering efficiency of the WGBL at room temperature for 785 nm excitation to be 4.5 ± 1 × 10^−9^ cm^−1^·sr^−1^, at a Stokes shift of 200 cm^−1^. This efficiency decreases monotonically for higher Stokes shifts. Additionally, we also demonstrate the use of slotted waveguides and quasi-transverse magnetic polarization as some mitigation strategies.

## 1. Introduction

**Figure 1:**
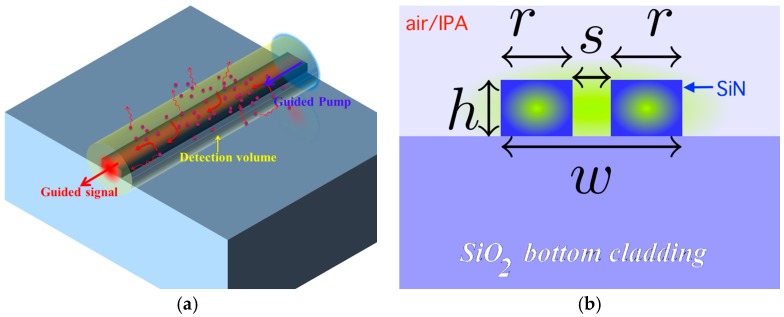
(**a**) Illustration of the nano-photonic waveguide enhanced Raman spectroscopy (NWERS) principle. The analyte is dispersed in the uppercladding, which is excited evanescently by a guided pump. The spontaneous Raman signal is collected by the waveguide evanescently. Adapted from [4], with permission from © 2016 The Royal Society; (**b**) A schematic of the cross-section of a generic silicon nitride (SiN) waveguide discussed in this paper. Slot waveguides are characterized by *s* > 0 while strip waveguides have *s* = 0. The top cladding (in light blue) may consist of Air, SiO_2_ as in Figure 2d, or an arbitrary analyte for Raman sensing.

Application of complementary metal oxide semiconductor (CMOS) technology for fabrication of photonic circuits has led to the emergence of silicon photonics, which has subsequently steered the innovation of numerous sensing techniques [1]. Recently, CMOS-compatible silicon nitride (SiN) waveguides have been shown to have promising properties to implement microscope-less Raman spectroscopy in a lab-on-a-chip framework [2,3,4,5,6,7]. In these studies, an enhanced evanescent field near a long single mode waveguide is exploited to enhance the pump and the collected signal, as illustrated in Figure 1. A combination of a large detection volume and moderate field enhancement near the vicinity of high-index-contrast waveguides leads to a guided signal that is more than three orders of magnitude higher as compared to that of a confocal microscope for a 1 cm long SiN waveguide spiral [6,7]. Such an enhancement of the signal in this nano-photonic waveguide enhanced Raman spectroscopy (NWERS) technique contributes to a higher signal-to-noise ratio (SNR) compared to the dark-noise limited micro-Raman systems [5,6,7,8], irrespective of the shot-noise from the background emission originating from the waveguide itself. Further, the smallest possible étendue of the collected signal in a single-mode waveguide allows the smallest possible integrated spectrometers for a given spectral resolution. A high performance, low cost, and compact Raman sensor can, therefore, be integrated on a chip using the technologies of a CMOS-fabrication. Notice this technique is quite different compared to surface-enhanced Raman spectroscopy, which exploits a large field enhancement in a small detection volume [4,9,10], and the signal is typically collected using a high étendue optical microscope [4,5].

The background emission originating from the waveguide itself, the waveguide background luminescence (WGBL), was largely ignored in earlier studies because the focus was more on the signal collected by the waveguide, and also partly because this background could be removed by simple background removal algorithms for large analyte concentration [11,12]. However, the shot-noise originating from the WGBL is critical to determining the signal-to-noise ratio (SNR) of the collected signal, especially for low analyte concentration. In this article we report a systematic experimental study on the properties of the WGBL, aimed at understanding its origin, and consequently develop mitigation and removal strategies.

## 2. Results

In this section, we detail experiments performed in order to pinpoint the origin of the WGBL and suggest a mitigation strategy based on the design of the optical mode. In Section 2.1, we describe a typical spectrum and identify several features of the WGBL originating from the SiN waveguides deposited using a plasma enhanced plasma chemical vapor deposition (PECVD) method and fabricated using fluorine based chemistry, as reported in [13] and subsequently used for the NWERS experiments [2,3,4,6,7]. We decompose the WGBL spectrum into a component dominant at low vibrational frequencies, henceforth referred to as a low-frequency dominant component (LFDC), and other components consisting of various peaks due to specific molecular vibrations in the core. Section 2.2 is dedicated to demonstrating that the WGBL is dominated by the signal from the core, followed by Section 2.3 where we determine the strength of WGBL relative to that of Iso-Propyl Alcohol (IPA), a standard analyte used in on-chip evanescent Raman spectroscopy. In Section 2.4 we focus on the study of the origin of the LFDC and discuss the dependence of this component on the excitation wavelength and input power to rule out the possibility that this feature originates from fluorescence or nonlinearities in the waveguide. In Section 2.5 we propose and demonstrate that the WGBL can be reduced by using slotted waveguides and quasi-transverse-magnetic (TM) modes. A mathematical model to calculate the contribution from the core material relative to the top cladding is developed in the Appendix A and used throughout the article. Unless otherwise stated, the measurements are done with the setup detailed in [2,4], which is also briefly discussed in the Methods section. The PECVD SiN waveguide used in the current experimental studies has also been reported in [13] and is also briefly described in Methods section.

### 2.1. Different Raman Features in the WGBL

**Figure 2:**
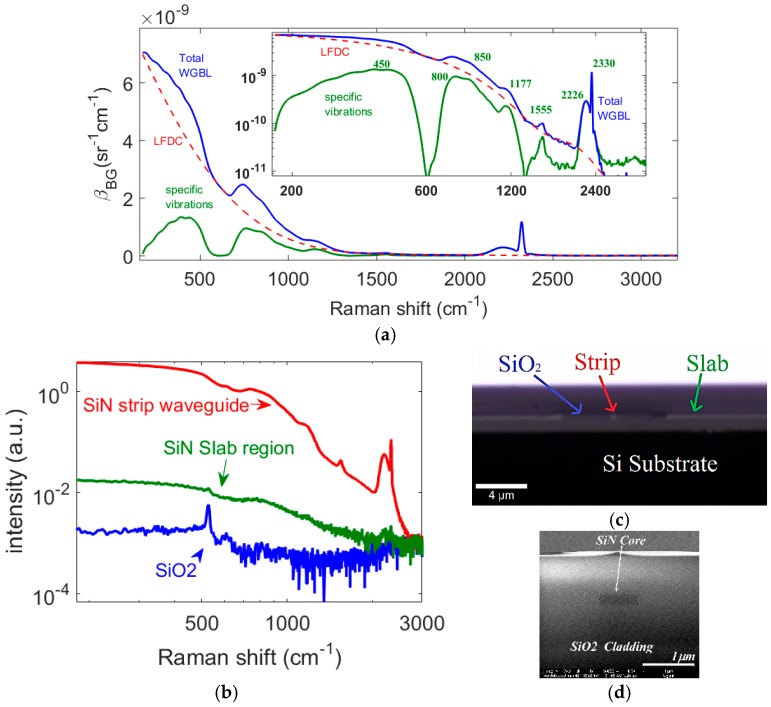
(**a**) The total waveguide background luminescence (WGBL) (blue solid line) from a 0.7 cm long, *w* = 700 nm, *h* = 220 nm, air-cladded strip waveguide measured using excitation and collection from the fundamental quasi-transverse electric (TE) mode, the low-frequency dominant component (LFDC) (red dashed line) and the LFDC subtracted Raman component (green solid line) ascribed to several chemical bonds in the core. The WGBL intensity spectrum is plotted in terms of Raman efficiency, *β*_BG_, for the entire Stokes region based on the value determined at 819 cm^−1^. In the inset, we also show the same spectra in log-log scale to cover the entire dynamic range of the spectrometer. The LFDC curve is based on a fifth order polynomial calculated using asymmetric cost function [11]; (**b**) Raman spectrum (red line) obtained using a confocal microscope by coupling the light into a strip waveguide (*h* = 220 nm, *w* = 700 nm) with SiO_2_ top and bottom cladding (see Figure 2c,d). The spectrum obtained from a slab region through the facet is shown in green. A confocal Raman spectrum (blue curve) obtained only from the SiO_2_ cladding region through its facet features a peak at 521 cm^−1^ from silicon, but several features contained in WGBL cannot be observed; (**c**) A microscope image of the facet used for confocal Raman spectroscopy shown in Figure 2b, the arrow indicates the region from where the spectra in Figure 2c were taken; (**d**) A SEM image of the strip waveguide core region.

In Figure 2a we display the WGBL intensity expressed in terms of the units of Raman efficiency, *β*_BG_ (see Appendix A for details), from an air-cladded, 0.7 cm long, 700 nm wide PECVD SiN strip waveguide measured using excitation and collection from the fundamental quasi-transverse electric (TE) mode collected using a setup that collects co-propagating spontaneous Raman signals coupled with the waveguide mode (see Section 4). The WGBL consists of a component that is monotonically decreasing with wavenumber shift and a combination of several convolved peaks. The earlier component, depicted by the red dashed line in Figure 2a, is referred to as the low-frequency dominant component (LFDC). These features, particularly the LFDC, are more distinguishable in logarithmic scale showing the WGBL intensity in the full dynamic range of the spectrometer. The LFDC is discussed in Second 2.4. In this paper we approximate LFDC with a 5th order polynomial using an asymmetric cost function [11].

In order to separate several peaks contained in the WGBL and identify their origin, we subtract the LFDC from the total WGBL. The subtracted spectrum is also shown in Figure 1a with a solid green line. The subtracted spectrum consists of features that have also been observed in the infrared absorption spectrum measured for silicon-nitride thin-films [14,15,16]. The major peaks centered around 450, 800, 850, 1175, 1555, and 2226 cm^−1^ are assigned, respectively, to Si breathing, Si-O bending, Si-N stretching, N-H bending, N-H_2_ bending, Si-H rocking and Si-H stretching [14,15,16]. The peaks due to hydrogen are suggestive of an incomplete reaction of hydrogen-rich silane, ammonia, and H_2_ during plasma enhanced chemical vapor deposition (PECVD). The strong narrowband peak at 2330 cm^−1^ is assigned to the interstitial N_2_ (typical diluent) incorporated in the core during the deposition process, as also reported in the deposition of GaN and ZnO [17].

At this point, it is instructive to look at the z-scan data of the SiN-SiO-Si stack presented in [18], which also suggests that several peaks are indeed due to the SiN rich waveguide core. To further corroborate the statement that the background peaks indeed originate from the SiN-rich core material and not SiO_2_ cladding, we measured the Raman spectrum of the stack at several locations of the waveguide cross-section depicted in Figure 2c,d using a confocal microscope (see Methods for the description of the measurement parameters). Figure 2b plots the confocal Raman spectra of the SiN-rich waveguide core material obtained by coupling the light into the strip waveguide, along with the confocal Raman signal obtained from the SiO_2_ cladding. The confocal spectrum from the strip waveguide, as well as from the slab region, feature most of the distinct features of the WGBL measured using the conventional NWERS setup, while most of these features are absent in the Raman spectrum of the SiO_2_ cladding. However, due to waveguide enhancement, the contribution from the strip waveguide is larger by more than two orders of magnitude compared to that of the slab region because of diffraction. All of these spectra are taken under similar experimental conditions including integration time and pump power. Hence, relative amplitudes from each location are indicative of their relative contributions for the WGBL. Thus, we conclude that the peaks contained in WGBL mainly consist of Raman peaks originating from the vibrations of several chemical bonds in the SiN waveguide core material and impurities incorporated therein during the deposition of the core material. This observation emphasizes the importance of the optimization of the deposition method in order to avoid incomplete reaction and the incorporation of the associated reaction products in the core.

**Figure 3:**
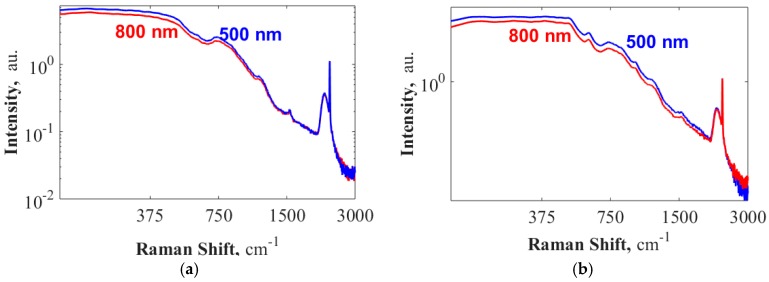
Difference in the spectra of the WGBL when normalized with the peak at 2330 cm^−1^ originating from the core. Excitation and collection for (**a**) TE polarization; and (**b**) Quasi-transverse-magnetic (TM) polarization using waveguide of widths *w* = 500 nm and *w* = 800 nm. A minor difference in the amplitude of the spectra (maximum: 0.8 dB) is observed.

Having confirmed that the origin of the several peaks observed in WGBL are from the core, next, we study the relative contributions of the core and cladding material for the LFDC. To this end, as shown in Figure 3a,b, we collect the WGBL spectra from waveguides with SiO_2_ as bottom and top cladding with different widths, namely *w* = 500 nm and *w* = 800 nm, and two orthogonal excitation and collection polarizations (quasi-TE and quasi-TM) that interact very differently with the cladding material [3]. We normalize these spectra with the peak at 2330 cm^−1^, as we know that it originates from the core. This normalization using an intrinsic peak allows us to get rid of the experimental differences, for example, that might occur due to differences of coupling and waveguide losses which vary from one measurement to another. In addition, assuming that the waveguide loss spectra for different Stokes shift is the same for these different cases, the use of a peak known to originate from the core renders the measured spectra relative to the contribution from the core for each of the different cases. As shown in Figure 3a,b, for a given polarization, we observe that except for a monotonously increasing amplitude difference between the two waveguide geometries, which culminates at about 0.8 dB at 200 cm^−1^, there is no significant difference in the spectra. We ascribe this difference to substrate leakage which monotonously increases for the longer wavelength for the smaller waveguide [13], particularly in the coupling section terminating at 220 nm.

On the ground of these arguments, and other experiments to be described in the next section, we can conclude that the SiO_2_ cladding does not have a significant contribution to the observed WGBL. In the Appendix A, we develop a theoretical model assuming that the WGBL is primarily due to core material. In the next section, we investigate the implications of this model in more detail with the aid of some experiments which further corroborate the conclusion derived here.

### 2.2. Dominant Source of the WGBL

In order to study the properties of the WGBL with the knowledge of the reference Raman signal from an analyte drop-casted as the top cladding, we quantify the signal-to-background ratio (SBR), *R*_S/B_ = *C*_s_*/C*_B_ as the ratio between the signal counts *C*_s_ from the top-cladding and WGBL counts *C*_B_ from the core measured at a given wavenumber. The implicit assumptions that are needed to treat the SBR *R*_S/B_ as a parameter of investigation are detailed in the Appendix A. Besides comparing the strength of the WGBL relative to a known analyte, the rationale for taking this ratio is to remove any common-mode signal variations across different measurements, such as differences in the coupling losses, waveguide losses, etc. In the present study, *R*_S/B_ is the ratio between the average signal counts of the 819 cm^−1^ Raman peak from the IPA upper-cladding and the average background signal evaluated at 819 cm^−1^. We investigate how *R*_S/B_ varies with waveguide length, waveguide width and mode polarization. This study will allow us to corroborate the suggestion from Section 2.1 that a significant amount of the WGBL, including the LFDC depicted by the red dashed line in Figure 2a, originates from the waveguide core.

**Figure 4:**
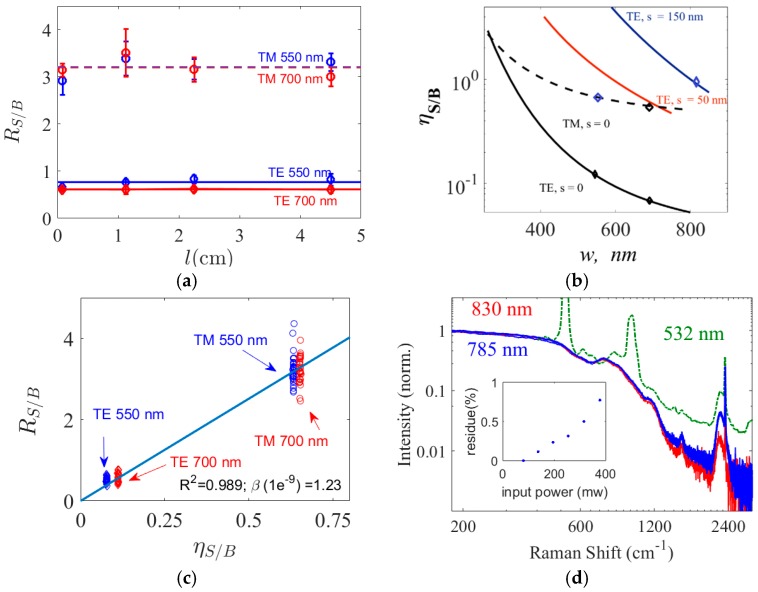
(**a**) The ratio of signal to the WGBL counts (*R*_S/B_) as a function of length evaluated at 819 cm^−1^ corresponding to an isopropyl alcohol (IPA) peak. We observe that *R*_S/B_ remains practically a constant. The blue lines are for a 550 nm wide waveguide and the red lines are for a 700 nm wide waveguide, while dashed lines are evaluated for the M polarization and solid lines for the TE polarization; (**b**) Calculated ratio of signal to the WGBL conversion efficiencies, *η*_S/B_, as a function of waveguide width for SiN waveguides with *h* = 220. Diamond markers correspond to the widths investigated experimentally in Figure 3a; (**c**) Average ratio of signal to the WGBL counts (*R*_A/B_) plotted against the calculated *η*_S/B_. The *R*^2^ of the fit is 0.989 and yields *β*_BG_ = 1.2 ± 0.1 × 10^−9^ sr^−1^·cm^−1^ at the Raman 819 cm^−1^; (**d**) The WGBL measured by exciting the waveguide at 532 nm (green dashed), 785 nm (blue), and 830 nm (red) wavelengths. The green dashed spectra are adapted from [18] and measured with a z-scan, hence they contain extra peaks at 520 and 960 cm^−1^ due to silicon. The blue spectrums contain an overlap of several spectra measured with pump powers ranging from 60 to 380 mW. The inset shows the residue, *Res*_i_, of the spectral difference observed relative to the spectra obtained using 60 mW.

In Figure 4a the dependence of *R*_S/B_ on waveguide length *l* is experimentally investigated for the two fundamental modes for the strip waveguides of thickness *h* = 220 nm and widths *w* = 700 nm and *w* = 550 nm. The experimental values are extracted from three chips measured three times. Two major conclusions can be drawn from Figure 4a. Firstly, the WGBL depends in a similar manner on waveguide length as does the Raman signal from the analyte. Secondly, we can conclude that the most significant contribution for the WGBL comes from the core of the waveguide, since the corresponding photon count ratio *R*_S/B_ is lower for the TE mode compared to the TM mode. It results from the fact that the confinement factor for the core (hence the corresponding photon counts contribution) is higher for the TE mode than for the TM mode for the given geometry of our waveguides where *h* ≪ *w* [3]. To further validate this statement, we calculate a parameter called *core-to-cladding conversion ratio* (CCR) *η*_S/B_. The CCR *η*_S/B_ is the ratio between the theoretical specific conversion efficiencies between the core and the top-cladding assuming the WGBL originates predominantly from the core, and is given by Equation (A8) in the Appendix A. The values of *η*_S/B_ calculated using COMSOL finite elements mode solver using this model are shown in Figure 4b for different waveguide geometries. As displayed in Figure 4b, a higher *R*_S/B_ is expected for the TM mode compared to that of the TE mode for both waveguides studied. Furthermore, as shown in Figure 4c, a linear fit between *η*_S/B_ and *R*_S/B_ for both TE and TM modes and both waveguide geometries with goodness of fit *R*^2^ = 0. 989 demonstrates a proportional relationship between *η*_S/B_ and *R*_S/B_. This is expected from Equation (A9), and indicates several assumptions made in the model are practically valid. As remarked in the Appendix A in the context of Equation (A9), the proportionality constant between *R*_S/B_ and *η*_S/B_ is the ratio between the scattering efficiencies (the product of the scattering particle number density and the scattering cross-section) of the WGBL and the signal. Hence, the data shown in Figure 4c can be used to calculate the background scattering efficiency *β*_BG_ relative to the top cladding scattering efficiency. This will be discussed next.

### 2.3. Determination of the Scattering Efficiency of the WGBL

Since the product of the concentration of pure IPA and the cross section of its 819 cm^−1^ line is known (*β*_s,819_ = *ρ*_s_ *σ*_s,819_ = 6.22 × 10^−9^ sr^−1^·cm^−1^) [2], the Raman scattering efficiency of the WGBL *β*_BG,819_ can be calculated to be *β*_BG,819_ = 1.2 ± 0.1 × 10^−9^ sr^−1^·cm^−1^ using the slope of the line fitting the data and Equation (A9). This value is about six times smaller than the value for pure IPA. Once *β*_BG_ is determined at one frequency, one can extrapolate the efficiency values to the entire Stokes region of interest by using a measured spectrum of the WGBL. The entire spectrum of the background efficiency, based on our measurement, is shown in Figure 2a. For an arbitrary analyte with a different cross-section or concentration (scattering efficiency), the value of the WGBL efficiency *β*_BG_ given in Figure 2a can be used to predict the signal-to-background ratio *R*_S/B_, or the corresponding SNR. In the last statement, we have implicitly assumed that all the features of the *β*_BG_ spectrum, including the LFDC, behave like a Raman signal. Next, we present some experimental observations and theoretical considerations, which show that this is indeed the case.

### 2.4. Low Frequency Dominant Component of the WGBL

In this section we focus on the study of the LFDC component of the WGBL that was shown with a red dashed line in Figure 2a. Generally, a broadband background emission in a Raman measurement is associated with auto-fluorescence arising from the electronic transitions of the material itself. The intensity of fluorescence depends strongly on the pump wavelength, while the spectral shape, plotted as a function of wavelength, does not change. Therefore, if plotted in *wavenumber shifts* with respect to different pump wavelengths, one observes a substantial change in the emission spectrum. A common strategy to discern fluorescence from Raman scattering, therefore, is to excite the material with wavelengths that differ by some significant amount (>500 cm^−1^ in wavenumbers) and then observe if the emission spectra in wavenumber shifts has changed. A significant difference in the spectrum means that the emission is primarily due to fluorescence. A Raman spectrum does not change since the Stokes spectrum shifts with the pump wavelength.

In Figure 4d, we show the WGBL (normalized to the maximum) generated by the fundamental TE mode of a 700 nm wide strip waveguide when excited at 785 nm and 830 nm wavelengths. The pump at 830 nm corresponds to about 690 cm^−1^ difference in wavenumbers compared to a pump at 785 nm. We observe a negligible difference in the spectra. A small difference that is observed is attributed to the difference in the quantum efficiency of the detectors for the two spectra in absolute wavelengths. The quantum efficiency of silicon detectors in the spectrometer at the Raman peak of 2330 cm^−1^ is lower for the 785 nm pump and even more so for 830 nm pump, as Si has a band-gap near to 1.14 eV (1087 nm). In Figure 4d, we also plot the spectrum obtained from a confocal Raman microscope using an excitation at 532 nm during a z-scan of the waveguide stack Si-SiO-SiN as reported in [18]. This spectrum is collected at the z-value corresponding to the strongest signal from the peak at 2330 cm^−1^ that originates from the interstitial N_2_ in the core material. The spectrum amplitude is normalized to the same scale for comparison. Apart from the peaks at 520 and 960 cm^−1^ ascribed to Si [18], which is unavoidable in a z-scan from the top of the material stack, there is no significant difference in the WGBL spectra. Thus, based on these measurements, we conclude that the WGBL spectra do not differ significantly over a pump wavelength spectral range between 532 and 830 nm. This conclusion excludes the possibility that a significant contribution of the LFDC originates from fluorescence of the waveguide material and indicates that it is due to Raman scattering.

However, a spectrum of emission due to a non-linear process, such as a *χ*^3^ process, may also be invariant to the pump wavelength to a certain degree. In order to rule out the possibility that the origin of the WGBL is a nonlinear process, we varied the pump power from 60 to 380 mW, (with ~8 dB in-coupling losses). In Figure 4d we have plotted all the WGBL spectra obtained from a strip waveguide of *w* = 700 nm and *h* = 220 nm for different input pump powers in blue (corresponding to a 785 nm pump wavelength). The normalized spectra show no visible difference. In order to quantify the variation of the total spectral shape for different input powers, we implemented a least-square algorithm that finds a normalization scalar $\phi_{i}$ that rescales the entire spectrum *S_i_*(*ν*) to a reference spectrum obtained using 60 mW input pump power *S*_60_(*ν*) such that the residue $Res_{i}=\sum_{\nu }{\left(S_{i}\left(\nu \right)-\phi_{i}S_{60}\left(\nu \right)\right)}^{2}$ is minimized. The rescaling of spectra using $\phi_{i}$ ensures that any difference in the entire spectral shape is quantified as *Res*_i_. In the inset of Figure 4d, the corresponding mean-squared error (*Res*_i_) is shown. The *Res*_i_ is less than 1% for the whole spectral range and all the input powers investigated. From this result we conclude that the shape of the WGBL does not depend on the input pump power level. The small and monotonic increase of the residue with the pump power can be attributed to the shot noise and the fluctuations of the input laser, which increases with the input power. If the WGBL originated from a non-linear process, the shape of the silicon nitride spectra should have been significantly influenced by the input power during the 6-fold increase in the waveguide power [19] thereby increasing the *Res*_i_ quite significantly.

In conclusion, in this section we ruled out the possibility that the LFDC observed at low wavenumbers in the WGBL is due to auto-fluorescence or any non-linear process. It is hence likely that it originates from a Raman scattering process occurring in the SiN core. Such broadband Raman scattering has been ascribed to breaking of momentum selection rules due to disorder in the amorphous material, and photon-phonon coupling due to thermal fluctuations [20,21,22]. A detailed study of the behavior of such a Raman scattering process in a guiding structure is outside the scope of this paper. The important conclusion from this section is that the LFDC efficiency spectrum *β*_BG_ calculated in Section 2.3 can be treated like a Raman spectrum. This conclusion is used in the next section to propose and demonstrate a mitigation strategy.

### 2.5. Mitigation Strategies

**Figure 5:**
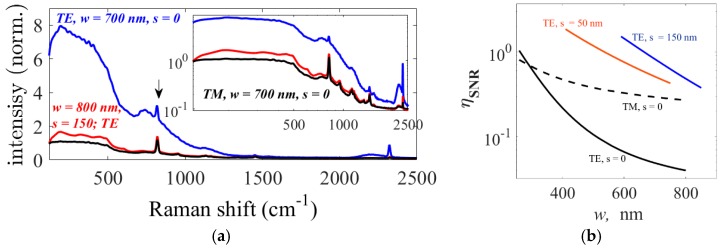
(**a**) The raw spectra of IPA measured using NWERS containing peaks from IPA as well as WGBL for *w* = 700 nm strip waveguide for TE (blue line) and TM (black line) polarization. The red spectrum for slotted waveguide, with *s* = 150, *w* = 800, and measured using the fundamental TE mode, is also shown. The spectra are normalized to the height of the IPA peak at 819 cm^−1^ (marked with an arrow) for comparison of their relative strengths; (**b**) Calculated value for signal-to-noise ratio (SNR)-equivalent efficiency for different waveguide geometries as a function of waveguide width.

In Section 2.2, a parameter called core-to-cladding conversion ratio (CCR) *η*_S/B_ was introduced using Equation (A8) in order to quantify the contribution of the waveguide core for the WGBL in comparison to the analyte signal from the top cladding. This parameter is plotted in Figure 4b for a core thickness *h* of 220 nm as a function of waveguide width for the TE mode of a slot waveguide (*s* = 150 nm) and both for fundamental TE and TM modes for strip waveguides. Evidently, the CCR *η*_S/B_ can be used to select the optimal mode and optimal waveguide design for high signal and low background, as the signal to background counts ratio (SBR) *R*_S/B_ is proportional to the CCR. For example, from Figure 4b, we expect high signal counts to background counts ratio (SBR) *R*_S/B_, for slotted waveguides compared to strip waveguides if both waveguides are excited using the fundamental TE mode. Similarly, the SBR for slotted waveguide is comparable to that of the fundamental TM mode in a strip waveguide. This is observed experimentally as already demonstrated in Figure 3a and further illustrated using their respective spectra in Figure 5a.

Although the CCR *η*_S/B_ is useful in determining the relative strength of the signal compared to the WGBL, it is not an accurate design parameter for optimization of the actual SNR. Standard deviation of the photon count fluctuation (shot-noise) due to the WGBL is actually proportional to the square root of the WGBL counts, since the photon count follows Poisson statistics. Hence, a more relevant design parameter is, *SNR-equivalent efficiency* *η*_SNR_ defined as:

(1) $$\eta_{\mathrm{SNR}}\equiv \frac{\iint_{Clad}\Lambda (\vec{r})d\vec{r}}{\sqrt{\iint_{Core}\Lambda (\vec{r})d\vec{r}}}=\frac{\eta_{S}}{\sqrt{\eta_{\mathrm{BG}}}}=\sqrt{\eta_{S}\eta_{S/B}}$$

Here, the numerator integral is evaluated in the top-cladding region where the analyte is located while the integral in the denominator is evaluated in the core region, which has been demonstrated to be the main source of the WGBL. Several quantities used in Equation (1) are further clarified in the Appendix A. The SNR-equivalent efficiency *η*_SNR_ is independent of analyte specific variables, such as analyte cross-section or density, and experiment specific variables, such as detector sensitivity. It is exclusively a function of the electromagnetic mode field distribution. Figure 5b shows the *η*_SNR_ calculated using Equation (1) for slot (*s* = 150 nm) and strip silicon nitride waveguides of various widths. Notice that one may derive a wrong conclusion if CCR *η*_S/B_ is used instead of *η*_SNR_ for design of the waveguide to minimize the SNR, as CCR does not account for the signal enhancement parameterized by *η*_s_ in Equation (1).

### 2.6. Remarks on the Wavelength Dependence of Several Experimental and Theoretical Parameters

In this paper, the influence of wavelength dependence of coupling efficiency, scattering efficiency, signal-to-noise ratio, and attenuation has been ignored. This leads to a negligible error in the conclusions and analyses we have presented for Stokes shifts <3000 cm^−1^ and waveguide widths >300 nm with cut-off wavelength >1030 nm (Raman shift ~3000 cm^−1^). As discussed in Section 2.1, substrate leakage at longer wavelengths contributes to a signal difference <1 dB for the largest Raman shift we have discussed, i.e., 3000 cm^−1^ [13]. Furthermore, ignoring Stokes shifts in the numerical model leads to a maximum of about 20% error (for Stokes shifts <3000 cm^−1^), which is comparable to the experimental error [3]. In addition, the setup used for the measurements described in this paper uses an achromatic lens to collect the Raman signal, hence there is negligible wavelength dependence of coupling of waveguide signal to the spectrometer. Consequently, the influence of wavelength dependence on these several factors has been ignored in this paper. An accurate determination of these influences may be needed if waveguide widths smaller than 300 nm or Stokes shifts much larger than 3000 cm^−1^ are of concern.

## 3. Discussion and Conclusions

The experiments described in this paper suggest that the WGBL originates primarily from the Raman scattering in the core, although a small component in the WGBL may contain the signal from the SiO_2_ bottom cladding or from auto-fluorescence from the waveguide materials. The experiments discussed in this article further suggest that the WGBL spectrum can be decomposed into two major components as discussed below.

(a) The WGBL consists of several peaks originating from the vibrations related to impurities incorporated during the deposition of the SiN core. This study is corroborated by the infrared absorption studies in the literature. It suggests that optimization of the deposition method is necessary to further minimize the incorporation of compounds contributing to several Raman peaks. Incomplete reactions during the deposition, especially those incorporating hydrogen based compounds and interstitial nitrogen in the core, should be minimized.
(b) The WGBL also consists of a slowly varying component that originates from Raman scattering due to thermal fluctuations of refractive index and momentum selection rule breaking in the amorphous core. The experimental studies with different pump wavenumbers, pump power, waveguide designs, and modes, as detailed in this article, strongly support this possibility. This also suggests that the use of crystalline materials may contract the WGBL to certain specific wavenumber shifts, thus, avoiding high SBR for a broad spectral range.

In conclusion, in this paper we identified the primary source of WGBL as the Raman scattering in the core of the waveguide. We also quantified the magnitude of the WGBL and demonstrated how the polarization of the fundamental modes or the modified geometry of the waveguides can be used to mitigate the effects from the background. The WGBL efficiency and method for its determination discussed in this paper will also be helpful in contexts other than Raman sensing, such as optical switching [23,24] and super-continuum generation [19].

## 4. Materials and Methods

### 4.1. Fabrication of the Waveguides

SiN waveguides used for the experiments discussed in this article were fabricated on 200 mm silicon wafers containing a stack of 2.2–2.4 μm thick high-density plasma chemical vapor deposition silicon oxide (SiO_2_) and 220 nm thick plasma-enhanced-CVD SiN. The structures were patterned with 193 nm optical lithography and subsequently etched by the fluorine based inductive coupled plasma-reactive ion-etch process to get the final structure [13].

### 4.2. Description of the Setup

A laser pump at 785 nm excites the waveguide via an aspheric lens. A half wave plate is used to rotate the polarization as needed to excite either TE or TM polarization of the waveguide. The analyte, which is typically pure isopropyl alcohol (IPA), is drop-casted on the waveguides and covers the relevant waveguide region. The Raman signal of the analyte is collected via the same waveguide. The signal is out-coupled using an achromatic objective, filtered using a long-pass filter, coupled to a single mode fiber using a parabolic mirror, and analyzed using a commercial spectrometer (AvaSpec-ULS2048XL, Avantes, Apeldoorn, The Netherlands) [2,4].

### 4.3. Confocal Microscope

A WITec Alpha300R+ confocal Raman microscope equipped with a Zeiss W Plan-Apochromat VIS-IR 100×/0.9 objective, a 785 nm excitation diode laser (Toptica, Munich, Germany), and an UHTS 300 spectrometer (WiTec, Ulm, Germany) using a −75 °C cooled CCD camera (ANDOR iDus 401, Belfast, UK) was used. A fiber with 100-μm diameter was used as a pinhole.

## Abbreviations

The following abbreviations are used in this manuscript:

CCD	Charge Coupled Device
CMOS	Complementary Metal Oxide Semiconductor
IPA	Isopropyl Alcohol
LFDC	Low Frequency Dominant Component
NWERS	Nanophotonic Waveguide Enhanced Raman Spectroscopy
PECVD	Plasma Enhanced Chemical Vapor Deposition
SiN	Silicon Nitride
SNR	Signal-to Noise Ratio
TE	Quasi-Transverse Electric
TM	Quasi-Transverse Magnetic
WGBL	Waveguide Background Luminescence

## Appendix A

The efficiency of excitation and the subsequent collection by a waveguide mode from of a particle located at a position ***r*_0_** can be described by a parameter called *integrated luminosity* Λ of the waveguide [3,4]. It is defined as the ratio of power *P_wg_* collected by the waveguide mode to the power of the pump *P_pump_* for a unit scattering over a cross-section *σ* of the particle:

(A1) $$\Lambda ({\vec{r}}_{0})=\frac{P_{wg}({\vec{r}}_{0})}{\sigma P_{pump}}$$

Neglecting the effects of the Stokes shift, Λ*_wg_* can be written in terms of the normalized modal electric field *e*(*r*) as [3,4],

(A2) $$\Lambda ({\vec{r}}_{0})=\frac{1}{n}{\left(\frac{n_{g}\lambda_{0}{\left|\vec{e}({\vec{r}}_{0})\right|}^{2}}{\iint_{\infty }\varepsilon (\vec{r}){\left|\vec{e}(\vec{r})\right|}^{2}d\vec{r}}\right)}^{2}$$

where *n*_g_ is the group index of the waveguide mode and n is the refractive index of the medium where the particle is located. For a random ensemble of incoherently scattering particles with a uniform volume density *ρ*, distributed in a volume *V*, the total power conversion efficiency can be calculated as follows.

(A3) $$\frac{P_{wg}(\omega )}{P_{pump}}\equiv \sigma (\omega )\rho \underset{V}{∭}\Lambda ({\vec{r}}_{0})d\vec{r}$$

We assume that the channel waveguide geometry is translationally invariant along the *z*-axis. Then, we can define a quantity called specific conversion efficiency *η* for a waveguide cross-section *S* in the xy-plane as,

(A4) $$\eta \equiv \frac{d}{dz}\underset{V}{∭}\Lambda ({\vec{r}}_{0})d\vec{r}=\iint_{S}\Lambda ({\vec{r}}_{0})d\vec{r}$$

such that the power *P_Tot_* collected by a lossless waveguide section of length *l* is given by,

(A5) $$\frac{P_{Tot}}{P_{pump}}=\eta \rho \sigma l$$

Thus, we define a specific conversion efficiency for the top-cladding region, where the analyte molecules that contribute to the signal are located, as follows.

(A6) $$\eta_{S}=\iint_{Clad}\Lambda ({\vec{r}}_{0})d\vec{r}$$

We assume that the WGBL originates in the waveguide core. Then, in a similar manner to that of Equation (A6), we can define a specific conversion efficiency for the core region to account for any WGBL emission from the core.

(A7) $$\eta_{B}=\iint_{Core}\Lambda ({\vec{r}}_{0})d\vec{r}$$

As the ratio of analyte signal conversion efficiency to the WGBL conversion efficiency, we quantify the core-to-cladding conversion ratio (CCR)

(A8) $$\eta_{S/B}=\frac{\eta_{S}}{\eta_{B}}=\frac{\iint_{Clad}\Lambda ({\vec{r}}_{0})d\vec{r}}{\iint_{Core}\Lambda ({\vec{r}}_{0})d\vec{r}}$$

We model the background originating from a waveguide as the Raman scattering signal originating from scattering particles in the core with scattering efficiency *β*_BG_. The scattering efficiency *β*_BG_ is equivalent to the product of the number density and the scattering cross section for the presumed scattering particles in the core. Thus, the signal to background ratio (SBR) *R*_S/B_ (the ratio between the signal counts from analyte and the counts due to the WGBL), can be written as follows using Equations (A5) and (A8):

(A9) $$R_{S/B}=\frac{\eta_{s}}{\eta_{B}}\frac{\rho_{S}\sigma_{S}}{\beta_{\mathrm{BG}}}=\eta_{S/B}\frac{\rho_{S}\sigma_{S}}{\beta_{\mathrm{BG}}}$$

In the experiments discussed in this article, we evaluate Equation (A9) by assuming that *β*_BG_ behaves like Raman scattering efficiency. Hence, the ratio *R*_S/B_ is independent of the pump power, waveguide length, waveguide attenuation, coupling losses, etc. This is a reasonable assumption if our assumption that WGBL originates from the Raman scattering in the core is true. In an experiment, *R*_S/B_ can be plotted against *η*_S/B_ for different waveguide geometries, waveguide-modes, and various experimental conditions. Note that in the model developed here we have neglected any contribution from the SiO_2_ bottom cladding to the background. A significant contribution from the bottom cladding adds a factor in the denominator of Equation (A9), which makes *R*_S/B_ a nonlinear function of *η*_S/B_ when plotted for different modes and different waveguide geometries. Hence, observation of a linear relationship between SBR *R*_S/B_ and CCR *η*_S/B_ for different modes and different waveguide geometries, as done in Section 2, validates our assumption that (a) *β*_BG_ is independent of the waveguide parameters, just like Raman scattering efficiency (*ρ_s_σ_s_*) in the cladding, and (b) the core is the dominating source of the background.
